# Effects of peanut ball use on perceived labor pain, fatigue, and mother’s perception of childbirth: a randomized controlled trial

**DOI:** 10.1007/s00404-024-07656-2

**Published:** 2024-08-05

**Authors:** Hacer Alan Dikmen, İlknur Münevver Gönenç, Ayşe Nur Ataş

**Affiliations:** 1 Faculty of Health Sciences, Department of Midwifery, Selcuk University, Selcuklu, 42250 Konya, Turkey; 2 Faculty of Nursing, Department of Midwifery, Ankara University, Ankara, Turkey; 3 Faculty of Health Sciences, Department of Midwifery, Selcuk University, Konya, Turkey

**Keywords:** Peanut ball, Labor pain, Fatigue, Perception of labor

## Abstract

**Purpose:**

The aim of this study was to evaluate the effects of using peanut balls on labor pain, fatigue, and the mother’s perception of labor.

**Methods:**

This is a randomized controlled intervention trial. Data were collected from the Intervention (peanut ball) and Control groups between July 2022 and June 2023, with 45 pregnant women in each group. Data were collected using a personal information form, the Visual Analog Scale-Pain (VAS-P), the Visual Analog Scale for Fatigue (VAS-F), and the Maternal Perception of Childbirth Scale (MPCS).

**Results:**

The VAS-P scores of the intervention group were statistically significantly lower than those of the control group 15 min after peanut ball application (*p* = .000). Immediately after and 15 min after peanut ball application, the mean fatigue score of the Intervention group was statistically significantly lower than that of the Control group (*p* = .000). There was no statistically significant difference between the mean duration of labor minutes in the two groups (*p* = .177). The mean MPCS scores of the intervention group and control groups were 62.73 + 7.30 and 47.17 + 9.12, respectively, and the difference was statistically significant (*p* = .000).

**Conclusions:**

The findings of this study indicate that the use of peanut balls during labor can effectively reduce labor pain and fatigue in pregnant women, without affecting the duration of labor. Additionally, the use of peanut balls has been shown to positively influence the perception of labor among pregnant women. Therefore, it is recommended that midwives educate pregnant women about the use of peanut balls during labor and provide support in their use.

## What does this study add to the clinical work

**Table:** 

Peanut balls are a safe tool for positioning pregnant women in labor. The peanut ball in labor reduces labor pain and fatigue in pregnant women. The peanut ball in labor positively affects the perception of labor of pregnant women.	

## Introduction

The birth ball is a tool that assists the fetus in establishing a more stable position within the pelvis and facilitates the progression of labor [1]. It is a non-invasive and non-pharmacological method that is frequently used in labor and delivery due to its cost-effectiveness [2]. Although the use of round balls at birth has been widely utilized since the 1990s, the use of peanut balls is more recent than round balls and is not as well known by health professionals as round balls [3].

Peanut balls are curved plastic balls that may be used as an alternative to round birthing balls [4] with different diameters according to the height of pregnant women (40–60 cm) [5, 6], with a peanut-shaped midline indentation [6, 7]. It allows pregnant women to assume lateral, supine, or sitting positions, facilitates pelvic opening, supports fetal rotation, descent, and progression of labor [4, 8], and can be safely used to support labor in pregnant women who must remain in bed during childbirth [5]. Additionally, studies have also reported that the peanut ball reduces labor pain [9, 10].

Labor pain is a unique experience for women [11]. It is among the most severe types of pain a woman will experience in her lifetime [12]. An increase in maternal perception of pain during labor may lead to a decrease in oxygen supply to the fetus. Pain may activate sympathetic nerves, causing an increase in catecholamines, decreased blood flow to the uterus, decreased cardiac output and blood pressure, and irregular uterine contractions [13]. Therefore, pain management in labor and providing adequate support is very important for the positive birth experience of the pregnant woman [14]. Changes in position can be used to reduce pain during labor [8, 15]. Peanut balls are a safe tool for positioning pregnant women in labor [10]. In their experimental study, Jayasudha et al. (2021) found that peanut ball use during the first stage of labor in primiparous mothers was an effective method of reducing pain perception [16]. A review of the literature reveals a paucity of studies on the use of peanut balls in labor pain management. Therefore, it is imperative to evaluate the efficacy of peanut ball use in this context.

Maternal fatigue is a prevalent symptom during labor and may have a deleterious impact on the course of labor [17, 18]. Fatigue is a symptom that is associated with the general health status of the pregnant woman. Increased fatigue may have a negative impact not only on maternal but also on fetal outcomes. Factors such as increased uterine contractions and synthetic oxytocin use during labor may increase maternal perception of fatigue [19]. Increased fatigue can lead to prolonged labor, maternal distress, and increased risk for postpartum hemorrhage [20]. The use of birth balls as a therapeutic and entertaining tool during contractions can help distract the mother, and thereby reduce fatigue [19]. Nevertheless, despite the acknowledged importance of this factor, there are no published studies that evaluate the effect of both round and peanut ball use on fatigue [19]. Given the potential benefits of the peanut ball on the birthing process, further research into its impact on fatigue is crucial to advance the existing body of knowledge.

The perception of birth is an important factor that can contribute to a positive or negative experience for the woman giving birth [21]. Childbirth is one of the most challenging psychological events in a mother’s life. Negative perceptions of childbirth can lead to psychological birth trauma (PBT), which can lead to post-traumatic stress disorder (PTSD) in women [22]. Such circumstances may give rise to a number of adverse outcomes, including deterioration in interpersonal relationships, difficulties in establishing a mother-baby attachment, a reduction in breastfeeding rates, depression, and an increased desire for a cesarean section [21–23]. A positive birth experience leads to a positive attitude towards motherhood, an increase in mother–baby relationship, and an increase in the mother’s self-esteem and satisfaction [23]. A mother with a happy birth experience has positive expectations regarding subsequent births [24]. One of the most crucial responsibilities of midwives and other healthcare professionals is to enhance the comfort and satisfaction of women during labor [1]. A systematic review concluded that the most effective strategies for creating a positive birth experience are “supporting the pregnant woman during labor” and “providing intrapartum care with minimal intervention” [25]. The use of a peanut ball is intended to reduce the physical and emotional discomfort associated with labor and delivery, as well as to enhance the positive birth experience by supporting the desired fetal position.

To date, no study has been conducted to assess the impact of peanut ball use on labor pain, fatigue, and maternal perception of labor collectively. Consequently, our study sought to evaluate the effects of peanut ball use in labor on pain, fatigue, and maternal perception of labor.

It is anticipated that this study will make a significant contribution to the existing body of literature on the subject. Currently, maternity schools in Turkey provide training on the use of birth balls during labor. However, the birth ball used in maternity schools is a round standard Pilates ball. Due to its spherical shape, the ball poses a risk to pregnant women, particularly in terms of potential slippage and falls. The peanut ball is a safer and more comfortable alternative to the round ball. Currently, the peanut ball is not used in maternity schools due to a lack of awareness among midwives. Therefore, the results of this study indicate that the use of the peanut ball in labor will likely increase.

### Research hypotheses

H1: Peanut ball use during labor affects pregnant women’s labor pain, fatigue, and perception of labor.

H1a: Pregnant women who use a peanut ball during labor have less labor pain compared to the control group.

H1b: Pregnant women who use a peanut ball during labor have less fatigue compared to the control group.

H1c: Pregnant women who use a peanut ball during labor have a positive perception of labor compared to the control group.

## Materials and methods

### Research design

**Fig. 1:**
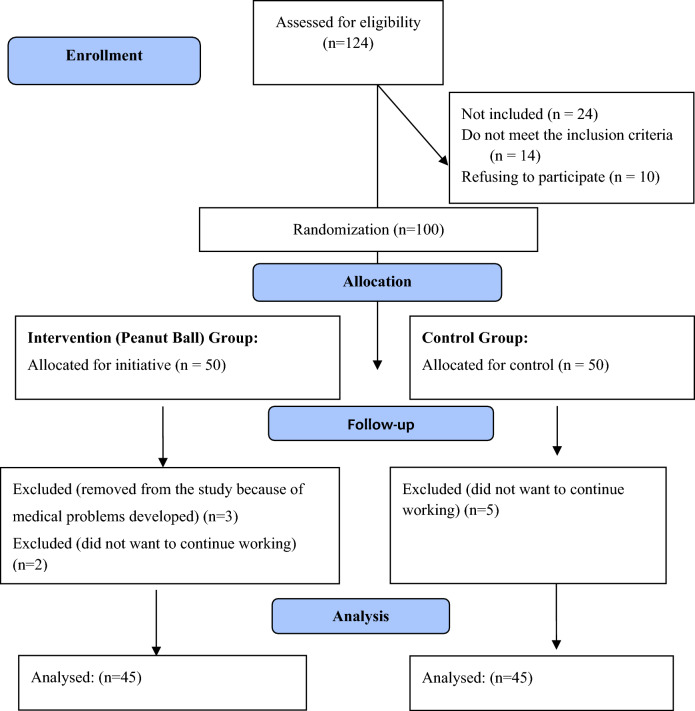
Flow diagram of the study

This is a randomized controlled intervention study. The reporting of the study adhered to the Consolidated Standards of Reporting Trials (CONSORT) guidelines [26] for parallel group randomized trials (Fig. 1).

### Place of research

The study was conducted in the delivery room of Necmettin Erbakan University Meram Medical Faculty hospital in Konya province, located in the Central Anatolia Region of Turkey. We chose this hospital because of the high number of pregnant women (average number of deliveries per month: 300–350) including patients from neighboring towns and districts. In addition, this hospital has the highest number of midwives and obstetrics and gynecology nurses in Konya province. In the hospital, pregnant women are admitted to single rooms for the duration of labor. The delivery of the infant is performed by both midwives and medical residents. In accordance with the standard operation procedure of the hospital, pregnant women are admitted to the delivery room, their medical history is documented in the admission unit, and they are introduced to the delivery unit. In the same unit, blood samples are taken for laboratory tests and intravenous catheters are inserted. Pregnant women are followed in the trauma department during the 1st stage of labor. Alternative positions, the plate ball, and the peanut ball are not used in labor. For the 2nd and 3rd stage of labor, the pregnant woman is transported to the delivery room, rest in the same room for 4 h after delivery, and then they are transferred to the postpartum ward.

### Population and sample of the study

Pregnant women admitted to the maternity clinic for delivery between December 2021 and December 2022 constituted the population of the study. Sample size was calculated using the G-power software program version 3.1.9.4. A priori power analysis was performed based on a fixed effects ANOVA test. The expected Cohens f value for effect size was 0.60, which is considered as a medium effect size. Therefore, it was determined that 45 pregnant women should be included in each group to reach 80% study power at 5% Type I error level with an effect size of 0.6 (medium). A total of 90 primiparous pregnant women were included in the study. At the end of the study, the power of the study was determined as 0.987 with a total of 62 pregnant women (31 participants in each group) (*α* = 0.05, Effect size *d* = 1.00), and the power of the study was determined as 1.00 (*α* = 0.05, Effect size *d* = 1.86) according to the power analysis according to the mean score of the Maternal Perception of Childbirth Scale.

### Inclusion criteria

Indication for vaginal delivery, cervical dilatation of 3 cm or more, term pregnancy (38–42 weeks of gestation), single, healthy, vertex positioned fetus, no complications that may cause dystocia in labor (contraction anomalies such as birth object, dystocia related to the birth canal, or dystocia related to the mother’s psychology), not using analgesia and anesthesia during the first and second stage of labor, not having any physical disability to take the positions to be used in the study, not having communication problems, and speaking and understanding Turkish.

### Exclusion criteria

Abnormal changes in fetal heart rate during labor (fetal distress, etc.), an unexpected complication in the pregnant woman or fetus, high-risk pregnancy, cesarean section, use of forceps or vacuum, taking magnesium sulfate, signs of intrauterine infection, previous attendance at a maternity school or training on birth balls, and withdrawal from the study.

### Participants

Pregnant women who were admitted to the maternity clinic to give birth and met the inclusion criteria were randomly assigned to the experimental and control groups.

### Randomization

Which group would be the Intervention Group (MG) or the Control Group (CG) was determined by drawing lots. The names of each group were written on pieces of paper and numbered according to the order of drawing. The intervention (peanut ball) group was numbered 1 and the control group was numbered 2. To mitigate the risk of data loss, random numbers between 1 and 100 were generated with the help of the Generate Numbers function on the Randomizer.org website [27]. Pregnant women were randomly assigned to one of two groups, designated as numbers 1 and 2 in the corresponding columns. Each woman was enrolled in the study individually to ensure that her participation would not influence the results of the other woman’s participation. The other pregnant woman was included in the study after the delivery process was completed.

### Data collection procedure

The data were collected between July 2022 and June 2023. Four data collection forms were used, and the data were collected in three phases.

Stage 1 (Admission to the clinic): a personal information form was administered to both the intervention (peanut ball) and control groups.

Phase 2 (Labor time): Both the intervention (peanut ball) group and the control group were administered the Visual Analog Scale (VAS) and the Visual Analog Scale for Fatigue (VAS-F).

Phase 3 (Postpartum): Both the intervention (peanut ball) group and the control group were administered the Maternal Perception of Childbirth Scale (MPCS) within the first 24 h after delivery.

### Personal information form

The personal information form, which was developed by the researchers based on the literature [5, 6, 28] and expert opinion, is comprised of two sections. The first section comprises ten items pertaining to some socio-demographic characteristics of pregnant women, including age, education level, marital status, social security status, and so forth. The second section comprises 11 items, including obstetric characteristics of the pregnant women (gestational week, desire for pregnancy, presence of complications during pregnancy, receipt of prenatal education, etc.).

### Visual analog scale for pain (VAS-P)

The VAS-P was developed by Price et al. [29] for the purpose of assessing pain. In the visual comparison scale, the patient indicates the level of pain on a ruler. This ruler is 10 cm in length. At one end of the ruler, the phrase “painlessness” is written and at the other end, the phrase “the most unbearable pain” is written. The use of this scale should be explained very well to the patient. The patient is told that there are two endpoints on the ruler and that they should mark the most appropriate place for the intensity of their pain in the plane between these two points. The distance between the point at which the patient indicates no pain and the actual location of the pain is measured in centimeters with a ruler and recorded [30]. In our country, the visual comparison scale has been used in numerous studies investigating the effect of non-pharmacological methods on labor pain management [31–33].

### Visual analog scale for fatigue (VAS-F)

The VAS-F was developed by Lee et al. [34]. The scale was adapted into Turkish, and its validity and reliability study was performed by Yurtsever and Bedük in 2003 [35]. The scale comprises a total of 18 items. Items 1, 2, 3, 4, 5, 11, 12, 13, 14, 15, 16, 17, and 18 pertain to fatigue, while items 6, 7, 8, 9, and 10 relate to energy subscales. The rows, each comprising ten items, are divided into two sections. The first section comprises negative statements, while the second comprises positive statements. Items that change from positive to negative are items of the fatigue subscale, while items that change from negative to positive are items of the energy subscale. While the lowest score obtained from the fatigue subscale is zero, the highest score is 130. The scores obtained from the energy subscale range from 0 to 50. Low scores on the energy subscale and high scores on the fatigue subscale indicate a high level of fatigue. The Cronbach’s internal consistency coefficient for the fatigue subscale was 0.90, while the Cronbach’s internal consistency coefficient for the energy subscale was 0.74. A high score on the fatigue subscale and a low score on the energy subscale indicate a high severity of fatigue [35]. In this study, Cronbach’s internal consistency coefficient of the energy subscale was 0.77, and Cronbach’s internal consistency coefficient of the fatigue subscale was 0.88. Permission to use the scale was obtained.

### Maternal perception of childbirth scale (MPCS)

The MPCS is an instrument that assesses how mothers perceive their experiences during normal or unplanned cesarean deliveries. The MPCS was developed by Marut and Mercer in 1979 as a 29-item scale [36]. In their factor analysis study in 1996, Fawcett and Knauth transformed the MPCS into a Likert-type scale with 25 items and 5 sub-dimensions. The sub-dimensions of the scale are as follows: “Experiences during labor” (7 items; 3, 5, 6, 8, 15, 17, 18), “Experiences during the pain period of labor” (7 items; 1, 2, 4, 7, 9, 10, 16), “Postpartum” (4 items; 22,23,24,25), “Partner involvement” (4 items; 11,12,20,21), and “Awareness” (3 items; 13,14,19). Each item in the Maternal Perception of Childbirth Scale is scored on a scale of 1 to 5, with 1 indicating “Not at all”, and 5 indicating “Very Much”. A questions 15-16-17-18-19 of the scale contain negative expressions, scoring should be done in the reverse direction for these questions [37]. The translation of the Maternal Perception of Childbirth Scale into Turkish and its validity and reliability study were conducted by Güngör and Beji in 2007. The Cronbach alpha value of the scale was 0.90, and the alpha values obtained with the two-half test method were 0.83 and 0.81 [38]. In our study, the Cronbach’s alpha value of the MPCS was 0.88. Prior to commencing the study, permission was obtained to use the scale.

### Preliminary application

To assess the comprehensibility and usability of the prepared data collection forms, a preliminary application was conducted with 10 pregnant women in the delivery room unit of Necmettin Erbakan University Meram Medical Faculty Hospital between July 16, 2022 and July 26, 2022. Following this preliminary application, no changes were made to the forms. The pregnant women who participated in the preliminary application were not included in the study.

### Interventions

#### Intervention group (peanut ball group)

A total of five positions were used in the study of pregnant women who received peanut ball application. Prior to the application, the peanut ball was introduced to the mothers by the researchers, and training was provided on the use of the peanut ball using a guide prepared by the researchers and developed with the input of three experts. The positions to be used with the peanut ball were demonstrated to the pregnant women in a practical setting.

The peanut ball was positioned between the pregnant woman’s legs and her back was supported with a pillow, and her position was changed periodically [5, 6, 39]. In addition to the peanut ball, pregnant women may be positioned in a variety of ways, including left side lying, right side lying, supine position, semi-sitting position, hand-knee position, squatting, and pushing positions [40]. In this study, the side-lying, supine, semi-sitting, hand-knee, and squatting positions were used. The dimensions of the peanut ball should be compatible with the dimensions of the pregnant woman. Otherwise, it causes strain on the joints. Peanut balls are available in four sizes: 40, 50, 60, and 70 cm. The most commonly used peanut balls are those of 40, 45, and 50 cm. A 40 cm peanut ball was used for women with a height of 160 cm or below, a 50 cm peanut ball for women between 161 and 169 cm, and a 60 cm peanut ball for women 170 or above or obese women. Only a 70 cm peanut ball is used in seated positions.

In this study, cervical dilation was not taken as a parameter. In the intervention group, VAS-P and VAS-F scores were taken before the intervention (when cervical dilation was 4 cm), at the end of the intervention (immediately after the intervention), and 15 min after the end of the intervention. The entire peanut ball intervention took 2–2.5 h for one woman. Data for the control group were collected as the first measurement when cervical dilation was 4 cm, the second measurement 2–2.5 h after the first measurement, and the third measurement 15 min after the second measurement.

In the intervention group, once the cervical opening reached 4 cm, the peanut ball was first applied to the pregnant women in the side-lying position (right side/left side) for 20 min. Thereafter, the pregnant women were allowed to rest for 10 min. Secondly, a semi-sitting position was applied for 20 min, after which they were allowed to rest for 10 min. Thirdly, the participants were placed in the supine position for 20 min, and after which they rested for 10 min. Fourthly, the hand-knee position was applied for 20 min, after which they rested for 10 min. Finally, the squatting position was applied for 20 min, after which they rested for 10 min. Each position was practiced with a peanut ball for 20 min, after which the participants rested for 10 min.

During the use of the peanut ball, the ball was encased in a sheath, which was replaced at each positional change of the pregnant woman. Five different sheaths were used for each pregnant woman. Following the use of the peanut balls in each pregnant woman, the sheaths were removed, cleaned, and disinfected through the application of a rapid surface disinfectant containing 50–60% ethyl alcohol. The peanut balls were prepared for use by affixing a new sheath.

#### Control group (CG)

Pregnant women in the control group received no intervention other than routine intrapartum midwifery care.

### Ethical permissions

Prior to the study, institutional approval was obtained from both the local research ethics committee (IRB: 2022/265) and the relevant hospital (Institutional Approval Number and Date: E-14567952-900-211427, 27.06.2022). The research was conducted in accordance with the ethical standards of the Declaration of Helsinki of 1964 and subsequent amendments or equivalent ethical standards. Participants who met the study criteria were informed of the study objectives and the benefits/risks of the peanut ball and written informed consent was obtained from all participants. Participants were also informed that they could withdraw from the study at any time for any reason, that their participation was completely voluntary, and that their identity would remain confidential.

### Data analysis

The data obtained in the study were analyzed using SPSS 20.0 (SPSS Inc., Chicago, IL, USA). Normality was assessed by calculating skewness and kurtosis values and using the Shapiro–Wilk test. Frequencies, percentages Chi-Square test, Fisher’s Exact test, independent samples t test, one-way ANOVA test, repeated measures ANOVA, and Pearson correlation analysis were used to analyze the data.

## Results

The socio-demographic characteristics of the participants are shown in Table 1. There was no difference between the groups in terms of socio-demographic characteristics (*p* > 0.05). There was no statistically significant difference between the intervention and control groups in the mean pain score before and immediately after the intervention (*p* > 0.005). The mean VAS-P score 15 min after peanut ball application was 7.53 + 1.12 in the intervention group and 8.58 + 1.15 in the control group, and the difference was statistically significant (*p* = 0.000) (Table 2). There was no statistically significant difference between the Intervention and Control groups in terms of fatigue and energy mean scores before the intervention (*p* > 0.005). Immediately and 15 min after peanut ball application, the mean fatigue scores of the Intervention group were 71.48 + 11.42 and 80.93 + 13.27, respectively, and the mean fatigue scores of the Control group were 82.46 + 15.05 and 97.88 + 13.98, respectively, and the difference was statistically significant (*p* = 0.000) (Table 3). The mean delivery time of the intervention group was 346 + 115.09 min and the mean delivery time of the control group was 388.24 + 173.79 min and there was no statistically significant difference between the two groups (*p* = 0.177). The mean MPCS score of the intervention group was 62.73 + 7.30 and the mean MPCS score of the control group was 47.17 + 9.12 and the difference was statistically significant (*p* = 0.000). The mean score of the Delivery Experience subscale for the intervention group was 23.64 + 3.29 and the mean score of the Delivery Experience subscale for the control group was 16.73 + 4.86, and the difference was statistically significant (*p* = 0.000). The mean score of the Labor Experience subscale was 24.06 + 2.72 for the Intervention group and 16.86 + 3.20 for the Control group, and the difference was statistically significant (*p* = 0.000). The mean score for the Delivery Outcome subscale was 18.97 + 1.13 for the Intervention group and 16.37 + 1.80 for the Control group, and the difference was statistically significant (*p* = 0.000). The mean score for the Awareness subscale was 9.86 + 1.99 for the intervention group and 8.33 + 2.09 for the control group and the difference was statistically significant (*p* = 0.000). The difference between the two groups in the mean score of the Partner Participation subscale was not statistically significant (*p* > 0.005) (Table 4).

**Table 1:** Socio-demographic characteristics of the participants (*n* = 90)

Socio-demographic and obstetric characteristics	Intervention group (*n* = 45)	Control group (*n* = 45)	Total	Analysis	
	Mean ± SD	Mean ± SD	Mean ± SD	*t*^a^	*p*
Age	22.84 ± 2.72	23.2 ± 3.47	23.02 ± 3.11	.540	.590
Duration of marriage	1.98 ± 1.09	2.27 ± 1.47	1.5 ± .50	1.052	.296
	*n* (%)	*n* (%)	*n* (%)	*χ*^2^^b^	*p*
Education level					
Primary school	12 (26.7)	18 (40.0)	30 (33.3)	2.000^b^	.368
High school	24 (53.3)	21 (46.7)	45 (50.0)		
University or higher	9 (20.0)	6 (13.3)	15 (16.7)		
Work status					
Working	6 (13.3)	4 (8.9)	10 (11.1)	0.450^b^	.502
Not-working	39 (86.7)	41 (51.2)	80 (88.9)		
Income perception					
Low	4 (8.9)	5 (11.1)	9 (10.0)	4.822^b^	.090
Moderate	38 (84.4)	30 (66.7)	68 (75.6)		
High	3 (6.7)	10 (22.2)	13 (14.4)		
Family type					
Nuclear	36 (80.0)	39 (86.7)	75 (83.3)	.720^b^	.396
Extended	9 (20.0)	6 (13.3)	15 (16.7)		
Social security					
Yes	38 (84.4)	40 (88.9)	78 (86.7)	.385^b^	.535
No	7 (15.6)	5 (11.1)	12 (13.3)		
Regular exercise before pregnancy					
Yes	13 (28.9)	14 (15.6)	27 (30.0)	.053^b^	.818
No	32 (71.1)	31 (68.9)	63 (70.0)		
Desire for pregnancy					
Yes	38 (84.4)	43 (95.6)	81 (90.0)	.157^c^	.079
No	7 (15.6)	2 ( 4.4)	9 (10.0)		
Having problems during pregnancy					
Yes	34 (75.6)	29 (32.2)	63 (70.0)	1.323^b^	.250
No	11 (24.4)	16 (35.6)	27 (30.0)		
Participation in prenatal training					
Yes	12 (26.7)	16 (35.6)	28 (31.1)	.829^b^	.362
No	33 (73.3)	29 (64.4)	62 (68.9)		

*SD* standard deviation

^a^Independent samples t test

^b^Chi-Square

^c^Fisher’s Exact Test

**Table 2:** Comparison of labor pain in ıntervention and control groups

Variables^a^	Intervention group (*n *= 45)	Control group (*n* = 45)	Analysis	
	Mean ± SD	Mean ± SD	*t*^b^	*p*
VAS-P 1	4.71 ± 1.29	4.11 ± 1.65	− 1.918	.058
VAS-P 2	6.09 ± 1.06	6.29 ± 1.40	.761	.449
VAS-P 3	7.53 ± 1.12	8.58 ± 1.15	4.349	**.000**
*X*^c^	79,595	87,138		
*p*	.000	.000		
Difference	1–2; 1–3; 2–3	1–2; 1–3; 2–3		

*SD* standard deviation

^a^VAS-Visual Analog Scale, VAS-P 1: Pre-Intervention, VAS-P 2: Immediately Post-Intervention, VAS-P 3: 15 min Post-Intervention

^b^Independent *t* test

^c^ANOVA with repeated measures

Bold values: *p* < 0.05 is statistically significance value

**Table 3:** Fatigue and energy comparison of ıntervention and control groups

Variables^a^	Intervention group (*n* = 45)	Control group (*n* = 45)	Analysis	
	Mean ± SD	Mean ± SD	*t*^b^	*p*
Fatigue 1	61.35 ± 14.03	61.35 ± 14.03	1.170	.245
Fatigue 2	71.48 ± 11.42	82.46 ± 15.05	3.389	**.000**
Fatigue 3	80.93 ± 13.27	97.88 ± 13.98	5.899	**.000**
*X*^c^	125.939	116.522		
*p*	**.000**	**.000**		
Difference	1–2; 1–3; 2–3	1–2. 1–3; 2–3		
Energy 1	25.71 ± 5.74	25.33 ± 5.96	− .306	.760
Energy 2	25.13 ± 5.68	18.84 ± 6.65	− 4.821	**.000**
Energy 3	22.86 ± 6.62	15. 75 ± 7.98	−4.597	**.000**
*X*^c^	101,939	90,152		
*p*	**.000**	**.000**		
Difference	1–2; 1–3; 2–3	1–3; 2–3		

*SD* standard deviation

^a^Fatigue 1 and Energy 1 Pre-Intervention, Immediately Post-Intervention (Fatigue 2 and Energy 2), 15 min Post-Intervention (Fatigue 3 and Energy 3)

^b^Independent t test

^c^ANOVA with repeated measures

Bold values: *p* < 0.05 is statistically significance value

**Table 4:** Comparison of maternal perception of childbirth scale and its sub-dimensions in ıntervention and control groups

Variables^a^	Intervention group (*n* = 45)	Control group (*n* = 45)	Analysis	
	Mean ± SD	Mean ± SD	*t*^b^	*p*
MPCS	62.73 ± 7.30	47.17 ± 9.12	− 8.923	**.000**
Delivery experience	23.64 ± 3.29	16.73 ± 4.86	− 7.890	**.000**
Labor experience	24.06 ± 2.72	16.86 ± 3.20	− 11.486	**.000**
Delivery outcome	18.97 ± 1.13	16.37 ± 1.80	− 8.190	**.000**
Partner participation	10.80 ± 4.12	10.75 ± 3.02	− .058	.954
Being aware	9.86 ± 1.99	8.33 ± 2.09	− 3.551	**.000**

*SD* standard deviation

^a^MPCS, Maternal Perception of Childbirth Scale

^b^Independent t test

Bold values: *p* < 0.05 is statistically significance value

## Discussion

In recent years, the peanut ball has emerged as a promising intervention for improving maternal and fetal health during labor. It is currently a common practice among midwives, and further studies are needed to investigate its effects on the labor process. In this study, it was aimed to evaluate the effect of peanut ball use on labor pain, fatigue, and mother’s perception of labor.

In the study, the mean VAS-P scores of the intervention group were significantly lower than those of the control group at 15 min after peanut ball application (3rd measurement). This finding supports hypothesis H1a. Labor pain is a frightening factor for most women and reduces their satisfaction with labor. There is evidence in the literature that the use of peanut balls during labor reduces labor pain [2, 5, 16]. The peanut ball may increase a woman’s mobility during labor, increasing her sense of control and reducing the need for analgesia [41]. The peanut ball, which is easy and safe to use, can be integrated into care practices by educating midwives and women’s health nurses about the use of the peanut ball to reduce labor pain and improve the birth experience for mothers during labor.

In this study, the mean fatigue scores of the intervention group were significantly lower than those of the control group at the 2nd (immediately after the intervention) and 3rd (15 min after the intervention) measurements, and the mean energy scores were significantly higher at the 2nd and 3rd measurements than those of the control group. This result shows that the use of peanut balls in labor reduces fatigue and increases energy levels in pregnant women, supporting hypothesis H1b. Fatigue, which is common in labor, is closely associated with pain and can have adverse maternal and fetal outcomes [2]. Therefore, pain and fatigue in labor should be evaluated and managed together. Peanut-ball exercises, which allow changes in position during labor, may be an effective exercise to relieve labor fatigue. Delgado et al. (2022) used a peanut ball for a total of 90 min of the active phase and had the pregnant women assume three different positions. As a result of the study, the mean fatigue scores of the pregnant women in the intervention group were found to be significantly lower than those of the control group. A meta-analysis study found that the use of peanut balls during labor reduced maternal fatigue [19]. The results of this study and the literature indicate that the use of peanut balls during labor reduces maternal fatigue. Making the mother feel more fit during labor can increase birth satisfaction and self-confidence. Integrative approaches such as peanut ball use during labor may provide an opportunity for early initiation of the mother-infant relationship in the postpartum period. Therefore, midwives and women’s health nurses should encourage mothers to use peanut balls during labor and teach them how to use peanut balls. The effect of peanut ball on fatigue and maternal–fetal outcomes should be determined in future randomized controlled trials and a protocol for its routine use in the delivery room should be developed.

In this study, there was no significant difference in the duration of labor, but the total duration of labor between the groups, but the total duration of labor the intervention group was 42.24 min less than in the control group. In the literature, the studies found that the use of peanut balls [42] and birth balls [43] during labor accelerated the progression of cervical dilation and shortened the duration of all stages of labor. Tussey et al. (2015) found a statistically significant decrease of 29 min in the duration of the first stage of labor and 11 min in the duration of the second stage of labor in the peanut ball group. Although there was no significant difference between the durations of the stages of labor in this study, the duration of the peanut ball shortening the stages of labor is similar to the findings of Tussey et al. (2015) [5]. One reason for this difference may be the smaller sample size in the current study compared to Tussey et al. (2015). Prolonged labor increases maternal fatigue and discourages her. For this reason, future studies could investigate the effect of applications that provide position change, such as the peanut ball, on labor duration with a larger sample size.

The mother’s physical and psychological comfort during labor is very important for a positive birth experience and perception. In addition, the mother’s freedom to move and change position during labor reduces negative perceptions of labor such as, pain and stress [42]. In this study, the positive birth perception of the intervention group was significantly higher than that of the control group. This finding supports hypothesis H1c. Similar to this study, Payton (2015) found that positioning intervention with peanut balls did not reduce the duration of labor, but it did receive positive feedback from pregnant women, especially in the second stage, and 71% of participants recommending the use of peanut balls [44]. Arslantas and Cankaya (2024) found that childbirth comfort and satisfaction mean scores between the groups were found to be statistically insignificant in their study [43]. In the study by Farrag and Omar (2018), 70% higher labor satisfaction was reported in the birthing ball group [45]. In parallel with this study, Baran (2021) found that the use of peanut balls during labor increased labor satisfaction in pregnant women [42]. With this Arslantas and Cankaya found that The use of birthing balls as a therapeutic and entertaining tool during labor can help distract the mother, reduce anxiety and fatigue, reduce pain, and consequently increase satisfaction with the labor process. Therefore, the use of peanut balls is recommended to increase the freedom of movement and position of pregnant women during labor.

## Conclusion

This study concluded that the use of peanut balls during labor reduced labor pain and fatigue in pregnant women, did not alter the duration of labor, and positively affected the perception of labor in pregnant women. In line with these findings, it is recommended that midwives and obstetric nurses should safely use peanut balls in labor and delivery to reduce labor pain and fatigue, to facilitate fetal descent, and help mothers have a more positive experience of labor. It is recommended that midwives and obstetric nurses, who play an active role in labor, be informed/educated about the importance of positions and movements with peanut balls in labor, how to use them, and raise awareness of them to practice in real life.

### Limitations and strength of the study

Because the study was conducted in a single hospital, the results cannot be generalized to the community. Another limitation of the study is that the intervention was administered by the researcher and she knew which group the participants were in. In addition, in this study, the small sample size and lack of information on chronic pain in pregnant women are other limitations this study. However, the strength of the study is that it is the first and only study to evaluate labor pain, fatigue, and perception of labor together. The study was designed as a single-blind randomized controlled trial and has applicable results in terms of clinical applications of peanut ball application and midwifery care in labor. The strength of the study is that the study population and sample consisted only of primiparous pregnant women and the effect of parity on birth outcomes was ruled out.

## Data Availability

The data that support the findings of this study are available on request from the corresponding author. The data are not publicly available due to privacy or ethical restrictions. Research data are not shared.
